# The role of smartphone app “WhatsApp” on achievement motivation and social intelligence among female undergraduate students

**DOI:** 10.1111/ppc.12582

**Published:** 2020-07-17

**Authors:** Mariam H. Alshaibani, Eman S. Qusti

**Affiliations:** ^1^ Department of Psychology Taif University Taif Saudi Arabia; ^2^ Department of Psychology Taif University Makkah Saudi Arabia

**Keywords:** academic motivation, social intelligence, WhatsApp

## Abstract

**Purpose:**

This study investigates the effects of using a smartphone application (WhatsApp) on achievement motivation and social intelligence in female students at Taif University.

**Design and Methods:**

The sample comprised 60 undergraduate female students from a college of education. The design was a quasi‐experimental nonequivalent (pretest and posttest) control group.

**Findings:**

Achievement motivation was significantly higher in the experimental group than the control group at posttest. However, no significant difference was found between the experimental and control groups in social intelligence at posttest.

**Practice Implications:**

Smartphones can increase student learning by fostering high‐level skills and concepts.

## 1 INTRODUCTION

The importance of social media continues to increase as more people gain access to the internet in Saudi Arabia. This is particularly the case for college‐aged students, who are likely the highest demographic in the country to both have a portable device or smartphone and actively use social media.^1^

Aljaad^2^ cited the example of the University of Jordan's Library, which adopted the use of social media and blogs to allow students and faculty members to post ideas, research papers, and articles. Faculty members also use social media to keep students up to date on course information. Aljaad^2^ also indicated that social media can complement current educational approaches and save time for both teachers and students. Based on Alamri's^3^ study, WhatsApp ranked number one as the most used social media app between college students, followed by Facebook.

A survey by Alabdulkareem^4^ showed that about 73% of students and 100% of teachers in that study used WhatsApp. WhatsApp was also shown to be used by female students for scientific research, especially when they were added to groups. The vast majority (about 95%) of the sample participants in related studies have social media accounts such as WhatsApp, Facebook, Twitter, and Snapchat.^5^ WhatsApp was the most preferred social media app used by university students in Saudi Arabia.^3^ “WhatsApp operates on nearly all current types of devices and operating systems used by higher education students. It can improve their achievement, performance, and the amount of information learned and the motivation of preparation with groups of different sizes” (Sayan,^6^ p. 88). Pratama and Kartikawati^7^ defined WhatsApp as “a mobile learning technology that can help students to learn social, easy to construct knowledge by sharing with other group members through short messages, and ease of online interactions quickly between learners and teachers” (p. 165).

Integrating WhatsApp into educational courses permits its users a variety of capabilities, such as making and developing content and sharing materials and views about online gathered knowledge.^8^

Teachers and students have been encouraged to use WhatsApp in education due to its positive qualities and up‐to‐date features that increase understanding.^9^

Many studies have indicated that social media in general has a beneficial role in learning and motivation,^1^, ^10^, ^11^, ^12^ as it can support traditional learning and increase students' success.^13^ However, there is still a need for more empirical studies concerning how these social media affect learning and other variables such as social intelligence.

Social intelligence denotes an individual's ability to establish successful relationships. Individuals who demonstrate social intelligence have the capacity to understand various social environments and handle each social situation appropriately.^14^ On the other hand, achievement motivation denotes an individual's internal capacity that triggers action. In the education sector, achievement motivation is of critical importance in empowering individuals to demonstrate willingness and enthusiasm regarding achievement of their learning goals.^15^ It is imperative to explore the real context of achievement motivation, which includes a person's key motivations for acting upon a certain standard.

Some researchers have sought to understand the existing relationship between social intelligence and achievement motivation. Social intelligence empowers learners to establish successful relationships with teachers and other learners that contribute positively to increasing the level of achievement motivation.^16^, ^17^ Being able to navigate social environments is a critical characteristic of social intelligence.

Modern day researchers have demonstrated that learners who have made significant achievements demonstrate remarkable social competencies. However, none of these studies have established a direct relationship between social intelligence and achievement motivation. There is evidence that the university setting fosters the development of social intelligence because it encourages the development of remarkable social skills (SS), positive attitudes, and functional relationships among learners.^18^, ^19^

However, there are limitations in the development of social intelligence within the university setting. On the other hand, students may exhibit achievement motivation that is not related to their levels of social intelligence. Therefore, future studies should embark on establishing a specific correlation between social intelligence and achievement motivation.

In this study we aimed to answer the following question:

What is the role of the smartphone app “WhatsApp” in achievement motivation and social intelligence among female undergraduate students?

The following null hypotheses were tested using statistical analysis:

‐There are no statistically significant differences at the level of *α* ≤ .05 between the mean scores of the experimental group and the control group in the posttest of achievement motivation.

‐There are no statistically significant differences at the level of *α* ≤ .05 between the mean scores of the experimental group and the control group in the posttest of social intelligence.

## 2 METHODS

The study used a pre and posttest quasi‐experimental, quantitative design using a control group and an experimental group.

This study had a quasi‐experimental design because the students were not randomly assigned to the class but were assigned independently of the study researchers.^20^ Both groups were expected to take the pre and posttest. Of the two, only the experimental group was exposed to the treatment. The researchers compared the effects of the study's independent variable (female students' WhatsApp use or nonuse) between the experimental and control groups. The dependent variables were assessed by traditional self‐report measures in both groups.

The study sample consisted of 60 undergraduate female students from the college of education at Taif University. Their median age was 22.1. A convenience sample was used. All participants were from the same department, particularly those pursuing Educational Psychology research methods, in the first semester of 2019.

A convenience sampling method was used, however, a prior sample size calculation using the G*Power program, assuming a medium effect size (D = 0.50) would be detected from the pre‐post measures of motivation and social intelligence between the two student groups and an *α* of .050 and power of 95%. The total sample size required was estimated to be 54 students. This sample size was adjusted with 10% of the base (nearly six students) added to the total sample to account for attrition and lost follow‐up cases. Therefore, the desired total sample size of students required was 60 female students divided randomly to each group.

The sample was exclusively female students due to two reasons. The first was due to the natural educational environment in Saudi Arabia. Female students are taught by female staff members in a separated environment, whereas the male students are taught only by male faculty members in a separate environment. The second reason was due to the fact that gender was considered a control variable to isolate its effects from the other dependent variables.

Before the paired sample *t* test was performed, the students' achievement motivation and social intelligence scores were compared in the pretest using an independent sample *t* test to ensure that they were equal. The results are shown in Tables 1 and 2. There were no significant differences between the two groups on the scales of achievement motivation and its dimensions (self‐confidence, fear of failure, perseverance, and ambition) or on the Social Intelligence Scale.

**Table 1:** Independent sample *t t*est between the mean scores of the experimental and control groups in the pretest of achievement motivation

Domain	Group	N	Mean	SD	*t*	*P*‐value
Self‐confidence	Control	30	19.33	2.023	0.272	.787
	Experimental	30	19.17	2.679		
Fair of failure	Control	30	18.70	2.215	−0.248	.805
	Experimental	30	18.87	2.933		
Persistence	Control	30	21.93	3.523	−1.350	.182
	Experimental	30	23.23	3.928		
Ambition	Control	30	22.70	3.816	−0.805	.424
	Experimental	30	23.53	4.191		
Total score of Achievement Motivation Scale	Control	30	82.67	8.636	−0.802	.426
	Experimental	30	84.80	11.743		

**Table 2:** Independent sample *t* test between the mean scores of the experimental and control groups in the pretest of Social Intelligence Scale

	Group	N	Mean	SD	*t*	*P*‐value
Total score of Social Intelligence Scale	Control	30	68.73	6.523	−1.647	.105
	Experimental	30	71.70	7.405		

### 2.1 Instruments

To achieve the goals of the study, and in accordance with the nature of the data and methodology, the researchers used the following.

#### 2.1.1 The Tromso Social Intelligence Scale

The Tromso Social Intelligence Scale (TSIS) was developed by Silvera, Martinussen, and Dahl^21^ to assess social intelligence. The TSIS is a self‐reported instrument that includes 21 items. The TSIS measures intelligence on the basis of three different subscales. The first subscale, social information processing (SIP), measures the ability to understand verbal or nonverbal messages regarding human relations, empathizing with and reading hidden messages as well as explicit messages. The second subscale, SS, measures basic communications skills, such as active listening, acting boldly, and establishing, maintaining, and ending relationships. The third subscale, social awareness (SA), measures the ability to actively behave in accordance with the demands of the situation, place, and time.

Each subscale comprises seven items. A 7‐point Likert scale is used for each item. The minimum and maximum scores in the items are 1 and 7, respectively. According to Silvera et al,^21^ the Cronbach's *α* internal consistency coefficient for SIP, SS, and SA were .81, .86, and .79, respectively. Regarding the validity of this study, experts were consulted, structural validity was assessed, and similar scale validity was applied to the original scale. Among 130 items in the item pool, 21 items that had factor values higher than 0.45 and correlations higher than 0.30 were selected. When varimax factor analysis was applied to these 21 items, three factors were found that corresponded to the theoretical basis. The SIS was translated from English into Arabic by following the forward and backward translation procedure.

To ensure the reliability of the questionnaire, it was applied to a sample of 50 students not included in the main study; Cronbach's *α* was used to measure reliability, which was established as .712.

#### 2.1.2 Achievement Motivation Scale

The researchers used a questionnaire assessing achievement motivation constructed on the basis of the literature and previous studies related to this subject. Based on the research, the achievement motivation theory by McClelland was considered to be the basis of all standards of achievement motivation. McClelland^22^ characterized achievement motivation as having a drive for success and being illustrated by effort and persistence in the face of challenges.

The researchers added the following descriptive definition: achievement motivation is the motivation that makes the student ambitious and persevering, based on a fear of failure, insistence on success, and high confidence that helps them reach their goals. The questionnaire consisted of 40 sentences divided into four domains or factors (ambition, perseverance, fear of failure, and insistence).

The instrument was designed to facilitate self‐reporting and measure the level of achievement based on 40 different variables influenced by four factors. A 4‐point Likert approach to the questions was used. There were four options ranging from 1 (never) to 4 (all the time). The lowest score possible was 40, while the highest possible score was 160.

### 2.2 Validity of the instrument

The content and face validity of the used questionnaires were assessed using a panel of experts (five persons). The panel was asked to assess the face and content validity of the questionnaires, to assess their applicability to the students in the contexts of achievement, motivation, mobile usage, social media, and so forth, and to indicate whether any of the items lacked fit with the intended measured by the pragmatic experiment. The resulting feedback showed that 80% of the panel members agreed on the validity of the scales. This was followed with a reliability analysis of the measured scales with the Cronbach's *α* test to ascertain whether the scales employed in the study were understood by students equally reliably.

### 2.3 Reliability of the instrument

To ensure the reliability of the questionnaire, it was applied to a sample of 50 students not included in the main study. Afterward, Cronbach's *α* was used to measure reliability. Results are shown in Table 3.

**Table 3:** Cronbach's *α* coefficient of each sub domain and composite reliability

Sub domain no.	Cronbach's *α*
1	.781
2	.849
3	.815
4	.874
Total score	.883

The results showed that reliability varied from subtest to subtest, and reliability coefficients ranged from .781 to .874 for the individual test scores. Composite reliability was established at .883.

### 2.4 Threats to research

We used the nonequivalent control group design, which uses nonrandom selection, because the class of research method at the College of Education already existed. According to Al‐Shareffeen and Al‐Kailani,^23^ the pretest can minimize the threat of internal validity, such as history, maturation, and instrumentation. On the other hand, the most important external threats, referred to in the study under limitation, were due to the convenience sampling that lead to the inability of generalizing the result of the study.

### 2.5 Usage of WhatsApp

An experimental WhatsApp group was created during the semester. Students were asked to voluntarily join the group. Next, the instructor explained the purpose and the benefits of the group. It was mentioned that the WhatsApp group was set up for academic purposes that would serve the students in numerous ways, such as accessing presentations and materials that are posted in the class, following up with class assignments, and tracking upcoming events such as midterm and final exams or upcoming quizzes.

Students participating through the WhatsApp group were also assigned to create questions and topics of discussions that related to the topic explained in class. Students were encouraged to participate by posting any audio or video materials from the internet that related or linked to the weekly subject. It was also explained to the students that the app can be used at their convenience as a tool for reaching the instructor and the other group members anytime and anywhere. Moreover, the instructor's office hours were effectively utilized through the WhatsApp group. This enabled the students to access the teacher at any time, who could, in turn, solve arising issues at the students' convenience.

Practically speaking, the group had weekly online meetings with the tutor who helped the members write scientific research projects. This was a requirement for the completion of the 15‐week course, with the aim of publishing the research through peer‐reviewed journals. Each week, the students were required to write about an element of the research besides learning how to search online through a different database. This was aided through audio and visual tutorials uploaded on the WhatsApp group. The students could access the videos and the instructor's voice messages.

The scientific research course contained theoretical subjects and practical applications. The theoretical concepts included the definition of research and its different methods, goals, elements of scientific research, how to choose a scientific research topic, and many other important concepts that pertained to the subject in question. On the other hand, the practical topics were related to writing a research paper and how online media could be used in researching various topics from various databases. The application of different scientific research methods for research was covered as well.

The class comprised both practical and theoretical sessions to cover the topic. The theoretical concepts related to educational scientific research were applied during the sessions. However, the practical concepts were applied through the use of WhatsApp.

The task of the first week's online meeting aimed at understanding how to register on the Saudi Digital Library (SDL). Students could select an interesting educational topic and then do research online for content about the subject through different SDL databases. The second online WhatsApp group meeting was about addressing the different educational research elements, such as the preface, study title, research problem, research hypothesis, operational definition, research assumptions, research limitations, literature review, procedures and method, methodology, references, and appendix. In this case, students were required to select a number of journals from the SDL database that addressed their selected topics and highlight and pinpoint the previous educational elements discussed. Thereafter, students were asked to analyze the content of the first elements of the educational research, which was the introduction from their selected journals, and to write an introduction to their own research project.

During the third meeting, students were asked to write about the third element and so forth. The final WhatsApp meeting was about searching online for possible options regarding journals for publication purposes and an overall conclusion of all the scientific research elements and submitting the research.

## 3 RESULTS

The following null hypothesis was tested using statistical analysis: There are no statistically significant differences at *α* ≤ .05 between the mean scores of the experimental group and the control group in the posttest of achievement motivation. The hypothesis was tested using independent sample *t* tests to assess the differences between the mean scores of the experimental and control groups in the posttest of achievement motivation. The results are shown in Table 4.

**Table 4:** Independent sample *t* test between the mean scores of the experimental and control groups in the posttest of Achievement Motivation Scale

Domain	Group	N	Mean	SD	*t*	*P*‐value
Self‐confidence	Control	30	19.70	2.366	−4.909*	.000
	Experimental	30	22.53	2.097		
Fair of failure	Control	30	19.50	2.968	−5.052*	.000
	Experimental	30	23.00	2.364		
Persistence	Control	30	22.73	3.08	−2.941*	.005
	Experimental	30	25.13	3.23		
Ambition	Control	30	22.37	2.97	−2.251*	.028
	Experimental	30	24.23	3.44		
Total score of Achievement Motivation Scale	Control	30	84.30	7.00	−5.125*	.000
	Experimental	30	94.90	8.90		

* The difference is significant at .05 levels.

Significant differences were found between groups in self‐confidence, fear of failure, perseverance, and ambition, with significantly higher scores in favor of the experimental group.

The second null hypothesis was tested as follows: There are no statistically significant differences at (*α* ≤ .05) between the mean scores of the experimental group and the control group in the posttest of social intelligence. The hypothesis was tested using independent sample *t* tests to assess the differences between the mean scores of the experimental and control groups on the SIS. Results are shown in Table 5. The result showed a significant difference in favor of the experimental group.

**Table 5:** Independent sample *t* test between the mean scores of the experimental and control groups in the posttest of Social Intelligence Scale

	Group	No.	Mean	SD	*t* test	*P*‐value
The total score of Social Intelligence Scale	Control	30	76.97	8.459	3.328*	.002
	Experimental	30	70.67	5.996		

* The difference is significant at .05 level.

## 4 DISCUSSION

Social media applications, especially WhatsApp, have become the most popular applications among university students in Saudi Arabia.^4^ The researchers tried to adapt this application to serve students in the learning process through a program designed for the purpose of this study.

The results showed that students' achievement motivation increased when they participated through WhatsApp. This result may have occurred because WhatsApp helped students to complete these educational tasks more effectively than traditional methods. This result was supported by many previous studies, such as those of Dar et al^9^ and Cetinkaya,^13^ that indicated the effectiveness of using WhatsApp in education in general. This finding is also in line with those of Jafarzadeh‐Kenarsari and Pourghane^24^ and Al‐Jreasee et al.^12^

The finding is also in line with those of Pratama and Kartikawati,^6^ which found that applying WhatsApp as an integrated mobile learning group investigation method in the learning process improves learning outcomes of students compared with face to face learning methods. Also, they found that social media like WhatsApp was capable of providing motivation for learners after integrated learning in science and technology.

Furthermore, Sayan^5^ stated that WhatsApp can be utilized in learning in the field of education. This may be explained by the fact that WhatsApp is an effective way for students to ask questions and, in return, find the correct answer, either from the teacher or other students. This can occur at any time, not only at the time of a specific lecture. In addition, the students' feeling that they are under the eye of the teacher and other students may motivate them to do their best and ask for the help they need, which may not be the case in the traditional lecture‐based atmosphere.

Social media tools enable students to become active participants in their learning process because of their interactive nature. This feature allows students to develop a learning experience with their instructors and peers. They also obtain resources and commentary from tutors in a way that avoids the intimidating feeling that some students experience when they have to ask questions in class and in front of their peers.^4^

The results did not show any changes in social intelligence, and this may have occurred because the program in this study was not long enough to foster the skill of social intelligence. Another factor could be that the social intelligence measure included movements of the body that are not reflected in the use of the WhatsApp; therefore, future researchers could use a different measure of social intelligence. It is also recommended to study other communities.

### 4.1 Strengths and limitations of the study

One of the strengths of the study is the use of social media, specifically WhatsApp for university students, because WhatsApp is a well‐known social media application among students. Several studies have been conducted about the use of WhatsApp in education; however, the researchers could not find studies that addressed the effect of using WhatsApp on social intelligence in students at Saudi universities. One of the limitations of the study concerns the use of self‐report measures, which may have introduced response biases. In addition, the researchers did not perform a follow‐up assessment for the experimental group.

## 5 CONCLUSION

In conclusion, this study's findings can be used to encourage educators to embrace the use of smartphone apps to improve academic achievement. The use of smartphone applications such as WhatsApp epitomizes asynchronous learning, which is beneficial since it increases student learning by enabling students to develop high‐level skills and concepts. It also reduces the probability of postponing learning activities and strengthens the student responsibility and self‐motivation.

Future studies should address other social network applications in relation to other variables, such as self‐efficacy. Furthermore, the program in this study can be applied in other countries.

### 5.1 Implications for nursing practice

The study indicated that the usage of WhatsApp application attributed to an increase in achievement motivation for university students. Hence, using WhatsApp can provide support in the teaching process for different educational purposes in several fields beside other platforms, especially nowadays in light of the global pandemic coronavirus disease‐2019 and the necessity of social distancing in education to curb the spread of the virus.
